# Identification of Thioaptamer Ligand against E-Selectin: Potential Application for Inflamed Vasculature Targeting

**DOI:** 10.1371/journal.pone.0013050

**Published:** 2010-09-30

**Authors:** Aman P. Mann, Anoma Somasunderam, René Nieves-Alicea, Xin Li, Austin Hu, Anil K. Sood, Mauro Ferrari, David G. Gorenstein, Takemi Tanaka

**Affiliations:** 1 Department of Nanomedicine and Biomedical Engineering, University of Texas Health Science Center at Houston, Houston, Texas, United States of America; 2 Institute of Molecular Medicine, University of Texas Health Science Center at Houston, Houston, Texas, United States of America; 3 Department of Biomedical Engineering, University of Texas Austin, Austin, Texas, United States of America; 4 Department of Experimental Therapeutics, University of Texas M. D. Anderson Cancer Center, Houston, Texas, United States of America; 5 Department of Bioengineering, Rice University, Houston, Texas, United States of America; 6 Department of Gynecologic Oncology, University of Texas M. D. Anderson Cancer Center, Houston, Texas, United States of America; 7 Department of Cancer Biology, University of Texas M. D. Anderson Cancer Center, Houston, Texas, United States of America; 8 Department of Pharmaceutical Sciences, Thomas Jefferson University, Philadelphia, Pennsylvania, United States of America; University of Hong Kong, Hong Kong

## Abstract

Active targeting of a drug carrier to a specific target site is crucial to provide a safe and efficient delivery of therapeutics and imaging contrast agents. E-selectin expression is induced on the endothelial cell surface of vessels in response to inflammatory stimuli but is absent in the normal vessels. Thus, E-selectin is an attractive molecular target, and high affinity ligands for E-selectin could be powerful tools for the delivery of therapeutics and/or imaging agents to inflamed vessels. In this study, we identified a thiophosphate modified aptamer (thioaptamer, TA) against E-selectin (ESTA-1) by employing a two-step selection strategy: a recombinant protein-based TA binding selection from a combinatorial library followed by a cell-based TA binding selection using E-selectin expressing human microvascular endothelial cells. ESTA-1 selectively bound to E-selectin with nanomolar binding affinity (K_D_ = 47 nM) while exhibiting minimal cross reactivity to P- and L-selectin. Furthermore, ESTA-1 binding to E-selectin on the endothelial cells markedly antagonized the adhesion (over 75% inhibition) of sLe^x^ positive HL-60 cells at nanomolar concentration. ESTA-1 also bound specifically to the inflamed tumor-associated vasculature of human carcinomas derived from breast, ovarian, and skin but not to normal organs, and this binding was highly associated with the E-selectin expression level. Similarly, intravenously injected ESTA-1 demonstrated distinct binding to the tumor vasculature in a breast cancer xenograft model. Together, our data substantiates the discovery of a thioaptamer (ESTA-1) that binds to E-selectin with high affinity and specificity, thereby highlighting the potential application of ESTA-1 for E-selectin targeted delivery.

## Introduction

Targeted delivery offers a significant advantage for the local delivery of therapeutic payload and/or imaging contrast agents to a specific target site and promises to improve the delivery efficacy to the target tissue while minimizing the exposure to normal tissues. Active targeting can be achieved through an interaction between surface molecules on the cells at pathological sites and their ligands conjugated to either the surface of delivery carriers or drug molecules. Numerous delivery strategies have been proposed for different target cell types *via* various cell surface specific ligands [1]. Vasculature targeted delivery has become an attractive strategy due to the phenotypic changes on the endothelial cell surface associated with pathological conditions such as inflammation and angiogenesis [2]. Therefore, identification of novel ligands that recognize pathological vasculature is of great interest.

The selectin proteins, E-, L-, and P-selectin, constitute a family of calcium-dependent cell surface glycoproteins that play a critical role in inflammation, mainly through recognition of specific carbohydrate ligands, sialyl Lewis X (sLe^X^) and sialyl Lewis A (sLe^A^) [3], [4], [5]. All three selectins share structural similarities and mediate the initial tethering and rolling of leukocytes to the endothelial wall [6]. Among the selectin family, E-selectin (CD62E, ELAM-1 or LECAM-2) has been highlighted as a potential therapeutic target based on its unique role in inflammation [4], [7], [8]. E-selectin is not constitutively expressed in endothelial cells, but is transcriptionally induced by NF-κB and AP-1 in response to inflammatory cytokines such as IL-1β and TNF-α [9]. Consequently, elevated E-selectin expression was reported in many types of inflammatory diseases including diabetes, atherosclerosis, rheumatoid arthritis, and cancer [10], [11], [12], [13]. In addition to E-selectin-mediated inflammation, many studies have suggested a potential involvement of E-selectin in the attachment and transmigration of circulatory metastatic cancer cells through the endothelium [14], [15], [16], [17]. E-selectin ligands including monoclonal antibodies, peptide, and carbohydrate ligand, have shown selective binding to the inflamed vasculature in both experimental animal models and clinical trials [18], [19], [20], [21]. However, medical applications of these ligands remain a challenge due to low affinity, low specificity, lack of serum stability, and immunogenicity [19], [22], [23]. Therefore, identification of a ligand with high affinity and specificity to E-selectin and favorable *in vivo* characteristics holds potential for effective inflamed vasculature targeted delivery.

Thiophosphate-modified oligo-nucleotide aptamers (thioaptamers; TA) are a new class of ligands that differ structurally from RNA and DNA and bind target proteins with high affinity (Kd: ∼ nM) and specificity [24], [25]. In addition, TAs offer significant advantages over conventional aptamers, peptides, small molecules or antibodies due to their unique chemical and biological properties: a) enhanced nuclease resistance [25]; b) easy synthesis and chemical modification [26]; and c) lack of immunogenicity [27]. Aptamers against P- and L- selectin have shown antagonistic activities against their respective targets, due to high binding affinities [28], [29]. However, no aptamers have previously been identified against E-selectin, despite its pivotal role in inflammation associated with multiple diseases. We have recently developed methods for combinatorial selection of TA libraries consisting of 10^14^ random sequences and have identified TAs that bind to a variety of target proteins [30], [31], [32]. In this study, we employed a two-step aptamer selection strategy, comprised of a recombinant protein-based selection from the library followed by a cell-based binding screening for the selection of a thioaptamer to E-selectin. Our data demonstrated that the E-selectin thioaptamer ESTA-1 binds to E-selectin with nanomolar affinity on cultured endothelial cells and tumor-associated vasculature in human and mouse carcinomas. These results suggest that ESTA-1 could be an attractive ligand for site-selective delivery of drugs and imaging agents to inflamed vasculature *via* E-selectin.

## Materials and Methods

### Ethics Statement

All animals were handled in strict accordance with good animal practice as defined by University of Texas Health Science Center Institutional Animal Care and Use Committee, and all animal work was approved by the committee (protocol # HSC-AWC-07-099).

### Reagents

Oligonucleotide primers were synthesized by Midland Certified Reagents (Midland, TX). The extracellular domain of recombinant human E-selectin (535 amino acid residues) was purchased from R&D Systems (Minneapolis, MN). Streptavidin-coated magnetic particles were purchased from Pure Biotech (Midlesex, NJ). Human microvascular endothelial cells (HMVECs) were a kind gift from Dr. Rong Shao (Biomedical Research Institute, Baystate Medical Center/University of Massachusetts at Amherst, Springfield, MA, USA). Anti-human CD31 antibody was purchased from BD Pharmingen (San Jose, CA). Anti-E-selectin antibody H18/7 was isolated from hybridoma purchased from ATCC (Manassas, VA) and used as a competitor of TA binding to endothelial cells. Anti-human E-selectin antibodies were purchased from Sigma (St. Louis, MO) and Innovex (Richmond, CA) and used for immunostaining for cultured cells and human carcinoma paraffin sections, respectively. Human carcinoma tissue array was purchased from US Biomax (Rockville, MD).

### Synthesis and isolation of DNA thioaptamer library

The synthesis of the DNA thioaptamer (TA) combinatorial library was described previously [24]. Briefly, a single-stranded DNA library (∼10^14^ different sequences) with a 30-nucleotide random region (N30) flanked by 23 and 21 nucleotide primer binding regions was chemically synthesized. The library (40 nM) was PCR amplified in a reaction containing Sp dATP(αS), dCTP, dGTP and dTTP (200 µM), MgCl_2_ (2 mM), biotinylated forward primer (5′biotin- CAGTGCTCTAGAGGATCCGTG-AC-3′) (300 nM), reverse primer (5′-CGCTCGGATCGATAAGCTTCG-3′) (300 nM) and AmpliTaq DNA polymerase (0.5 U). Biotinylated double-stranded PCR products were incubated with streptavidin-coated magnetic beads for the separation of the ssDNA library.

### Selection of thioaptamers

Screening of TAs that binds to recombinant E-selectin protein was carried out using a solution-based filter binding method as described previously [24]. Briefly, the recombinant human E-selectin protein (240 pmoles) was incubated with TA library (200 pmoles) in selection buffer (PBS with Ca^2+^ and Mg^2+^ and 5 mM MgCl_2_) at room temperature for 2 hours. The reaction mixture was filtered through the nitrocellulose membrane and washed 3 times with the selection buffer to remove unbound TAs. The TA/E-selectin complex retained on the filter membrane was eluted with 8 M urea solution. The eluent was used as the template for PCR amplification and the integrity of the TAs was analyzed by 15% polyacrylamide gel electrophoresis. This selection cycle was repeated 10 times and the stringency of the selection was elevated gradually. The TA libraries obtained after rounds 5 and 10 were PCR amplified and subcloned into a plasmid vector for DNA sequencing. The selected sequences were analyzed using the ClustalW program (with DNA identity matrix, gap open penalty of 15, gap extension penalty of 6.66, gap separation penalty of 4 with no end gaps). Cyanine 3 (Cy3)-labeled TAs were produced by PCR amplification of plasmids containing TAs as a template with 5′-terminal Cy3-labeled reverse primer.

### Cell culture

Human Microvascular Endothelial cells (HMVEC) were a kind gift from Dr. Rong Shao at the University of Massachusetts. HMVEC were cultured according to the protocol described previously [33]. HMVEC were grown in endothelial basal medium-2 supplemented with 10% (v/v) Tet-approved fetal bovine serum, 100 U/ml penicillin and 10 µg/ml streptomycin, 1 µg/ml epidermal growth factor and 50 µg/ml hydrocortisone. All experiments were performed on 70–80% confluent cultures in 5% CO_2_ humid chambers at 37°C. The HMVECs were genetically manipulated to generate Tet-on inducible system for E-selectin expression (ES-Endo). E-selectin expression was induced with doxycycline (2000 ng/ml) for 5 hours unless specified.

### TA binding to endothelial cells

To examine TA binding to the ES-Endo, the cells were plated onto a plastic dish and cultured overnight to allow them to attach. After E-selectin induction with doxycycline, the cells were incubated with cy3-labeled TAs at the indicated concentrations (0–200 nM) for 20 minutes at 37 °C. The cells were washed with ice-cold PBS to remove unbound TA and subsequently fixed with 4% paraformaldehyde for 10 minutes. The nuclei were counterstained with 1.0 µg/ml Hoechst 33342 for 10 minutes. The extent of TA binding to the cells was assessed by fluorescence microscopic analysis (TE2000-E, Nikon, final magnification 60x). The relative binding affinity of TAs was determined by the amount of fluorescence detected on the cells based binding assay and the specificity was determined by the extent of doxycycline dose dependent effect seen on TA binding. For competition of TA binding to the cells, the cells were pre-incubated with 10 mg or 25 mg of anti-E-selectin antibody (H18/7) for 2 hours prior to incubation with TA. All images were acquired under the same exposure conditions for the comparison of TA binding.

### TA binding to tumor vasculature

Human tissues derived from epithelial ovarian cancer patients were collected from surgical cases at The University of Texas M.D. Anderson Cancer Center. Frozen tissue arrays derived from human carcinomas (breast, ovarian, and skin) and their normal counterparts were also used (US Biomax, MD). The tissue sections were fixed with ice-cold acetone, incubated with 50 nM ESTA-1 for 1 hour at RT, and then stained with primary antibody against anti-rat CD31 (1∶1000). E-selectin expression was determined by immunostaining with anti-E-selectin (1∶20). For *in vivo* experiments a total of 10 µg of chemically synthesized Cy3-labeled ESTA-1 was intravenously injected into mice bearing tumors derived from mouse breast cancer 4T1 cells. The organs and tumors were harvested 3 hours after the injection, and each organ was embedded in OCT. 8 µm frozen section was fixed with acetone and stained with Hoechst 33342 for assessment of ESTA-1 binding to the vasculature.

### Electrophoresis mobility shift assay

Equal amounts of ESTA-1 (4.6 pmoles) were incubated with increasing concentrations of the recombinant selectin proteins (0–19 pmoles) in a total volume of 10.5 µl of PBS supplemented with Ca^2+^ and Mg^2+^, 5 mM MgCl_2,_ and 1% NP40 at room temperature for 45 minutes. The reaction mixtures were loaded onto 6% polyacrylamide tris borate gels and run at 100 V for 90 minutes at 4°C. The gel was stained with SYBR Gold nucleic acid staining dye and visualized using the FluorChem 8800 chemimager (Alpha Innotech). Protein-bound TA and unbound TA were quantified using ImageJ software. The binding curves were generated assuming a single binding site curve fits using the Graph Pad Prism software. For competition experiments, anti-E-selectin antibody (H8/17) (3 µg, 9 µl) or for control experiment human IgG antibody was mixed with recombinant E-selection protein (19 pmoles) and 1% NP40 in PBS (3.7 µl) and incubated for 30 minutes. To this mixture, ESTA-1 (4.6 pmoles) in PBS and 5 mM MgCl_2_ was added and incubated for another 10 minutes and loaded onto 6% polyacrylamide gel and ran at 100 V for 80 minutes, stained with SYBR Gold stain and visualized using the chemiimager.

### Cell adhesion assay

To determine the effect of ESTA-1 on adhesion of sLe^x^ positive cells to endothelial cells, confluent ES-Endo were incubated with doxycycline for 5 hours followed by ESTA-1 (50 nM and 100 nM) for 20 minutes. HL-60 cells (10^5^ cells) suspended in RPMI containing 1% FBS were added to ES-Endo and incubated at 4°C for 30 minutes with mild agitation. The unbound cells were washed off with RPMI containing 1% FBS. The number of cells that adhered to the ES-Endo was counted on at least 3 random areas using a light microscope (final magnification 100x) and expressed as the mean of triplicate experiments.

### Cell viability

ES-Endo were cultured on a 96-well plate at 10,000 cells per well. The cells were incubated with doxycycline for 5 hours and then incubated with ESTA-1 at the indicated concentrations for 48 hours. For the measurement of cell viability, 10 µl MTT (5 mg/ml) were added to each well and incubated for 4 hours. The formazan was dissolved in 150 µl of DMSO and the absorbance at 490 nm was measured.

### Statistical analysis

All experiments were carried out in triplicates and the data were analyzed statistically to provide 80% power for a test at significance level of 0.01. We validated the normality assumption, and proceeded with a parametric test as appropriate. The Student-T test was performed to compare the cell viability among different groups.

## Results

### Screening of TA against E-selectin

We screened a thioaptamer (TA) library to select for those TAs that demonstrated affinity for E-selectin. Each of the 10^14^ TAs in the library consisted of a region of random sequence (N_30_ residues) flanked by two primer regions common to all TAs, and all dA's contained 5′-monothiophosphate substitutions with the exception of the 5′ primer region. A two-step E-selectin TA selection strategy followed. First, a solution-based combinatorial selection method was employed for the identification of thioaptamers that bind the extracellular domain of recombinant human E-selectin protein. The TA library was allowed to interact in solution with glycosylated recombinant E-selectin protein. Then, the E-selectin/TA complexes formed were isolated and PCR amplified to be used in subsequent cycles of selection. After 10 iterative selection cycles, 35 TA sequences were isolated. Sequences were aligned using the ClustalW program based on their primary sequences (Fig S1), and a cladogram tree was generated. Based on the alignment and the cladogram, the 35 sequences were grouped into 14 families (Fig S2). A single sequence was selected from each family based on the lowest predicted free energy of the secondary structure. Several common sequence motifs were identified among the selected 14 sequences (Fig S3). These 14 TA sequences were synthesized by PCR with Cy3-labeled reverse primer for the second step of cell-based selection.

**Figure 1:**
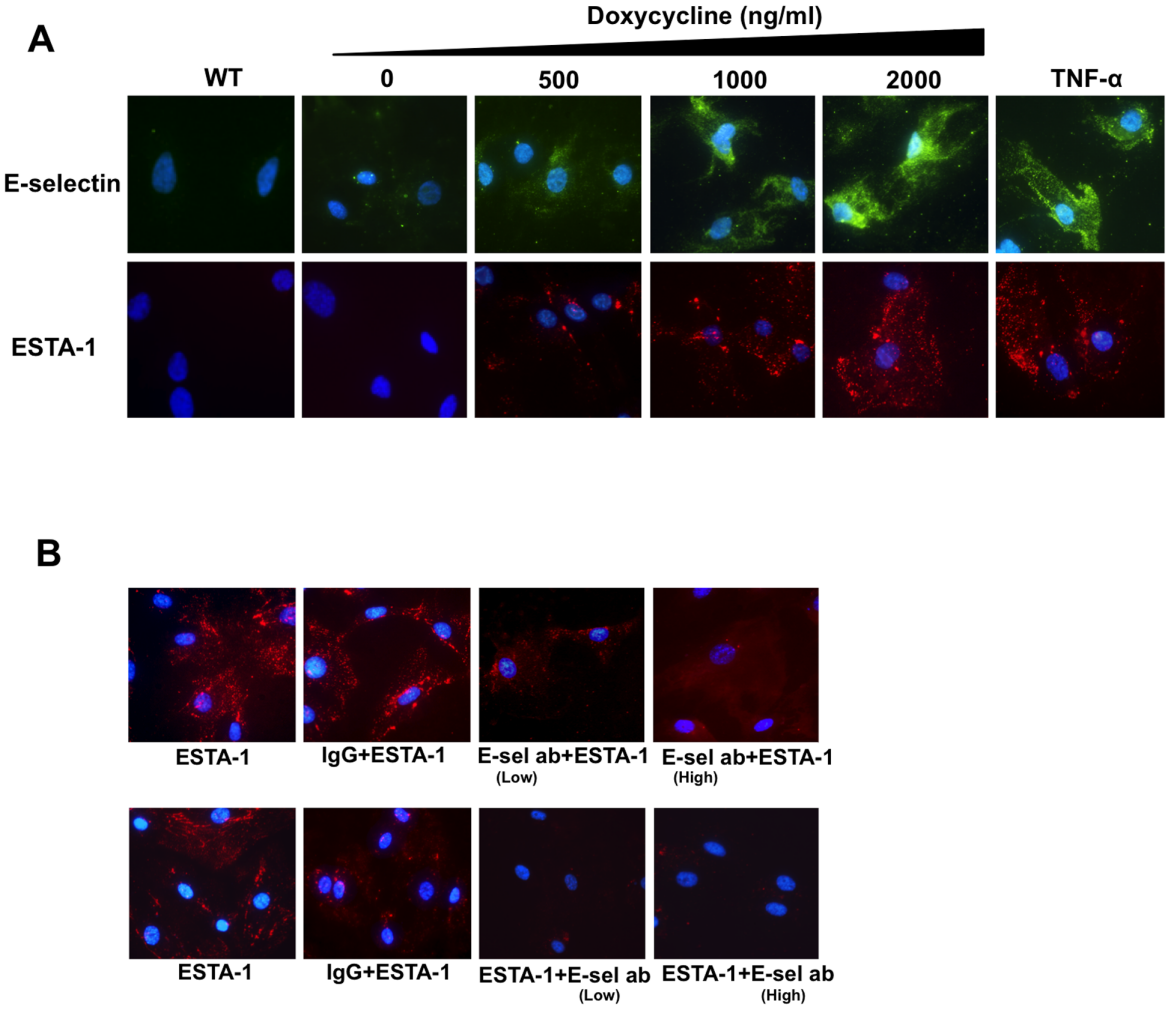
E-selectin dependent binding of ESTA-1. (A) ESTA-1 binding to ES-Endo. To determine the E-selectin dependent ESTA binding, ES-Endo cells were treated with increasing concentration of doxycycline (250-2000 ng/ml) and analyzed for E-selectin expression and ESTA-1 binding. E-selectin overexpressing ES-Endo cells were incubated with Cy3-labeled ESTA-1 (100 nM) for 20 minutes at 37°C. TNF-α (10 ng/ml) induced ES-Endo was used as a positive control. (B) Blocking of ESTA-1 binding by E-selectin antibody. ES-Endo were pre-incubated with 25 µg of E-selectin antibody for 2 hours and incubated with 100 nM of ESTA-1 for 20 minutes. Unbound ESTAs were washed away and slides were prepared for fluorescent imaging to visualize the binding to ES-Endo cells. All images were captured at the same exposure condition for comparison. The final images shown are representative images (at the final magnification: x600) from five random fields of at least three independent experiments. Blue, Hoescht 33342; Red, Cy3-labeled ESTA-1; Green, E-selectin.

**Figure 2:**
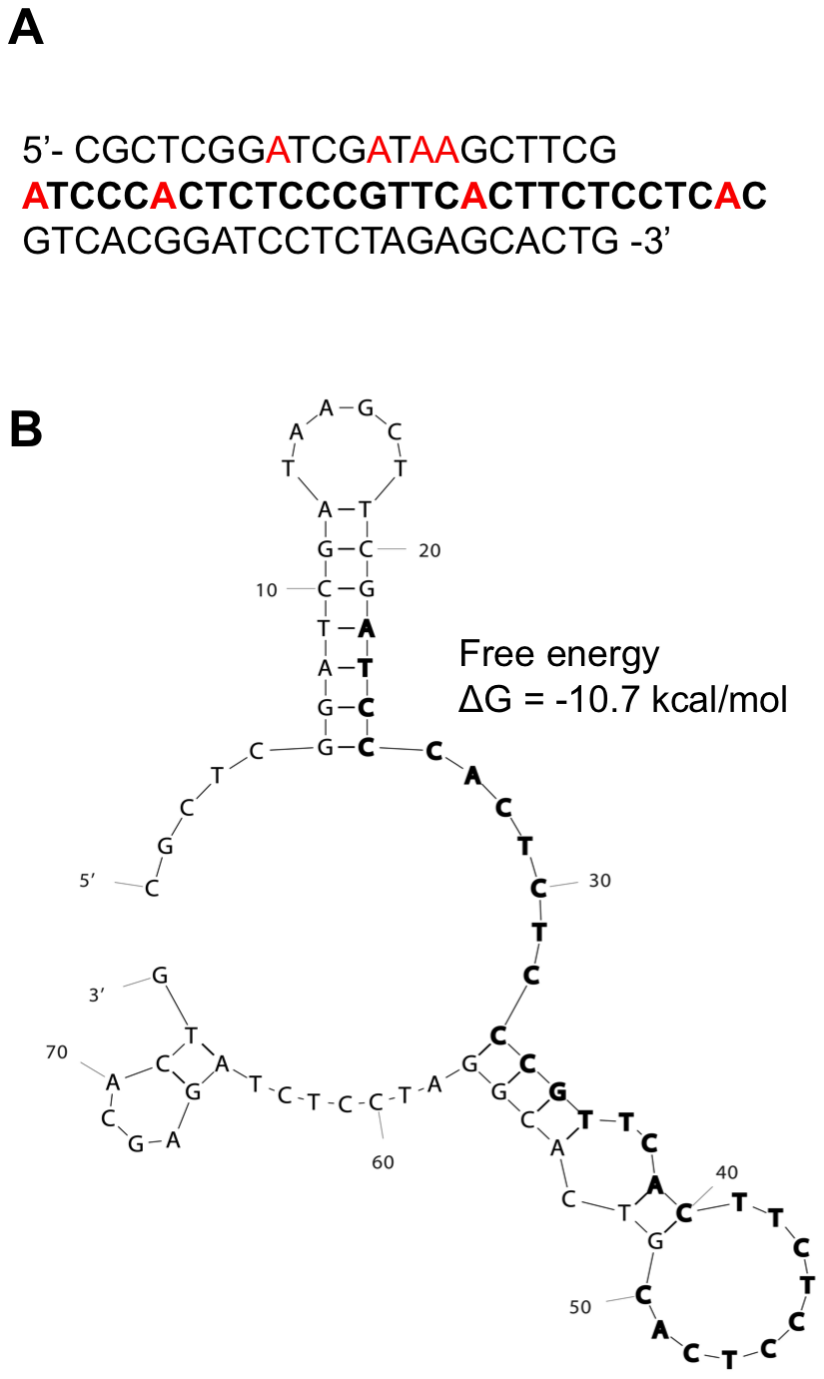
ESTA-1 sequence and the MFOLD predicted secondary structure. (A) ESTA-1 DNA sequence. All of the deoxy adenosine (dA) residues are modified monothio substituted with Rp configuration, with the exception of the 5′-primer binding region in the sequence. (B) Mfold predicted secondary structure of ESTA-1.

A Tet-on inducible E-selectin endothelial cell line (ES-Endo) was used to identify the TA sequences that specifically bind to E-selectin on the surface of endothelial cells. First, to demonstrate the doxycycline-dependent induction of E-selectin expression, ES-Endo cells were incubated with increasing concentrations of doxycycline (0–2000 ng/ml) for 5 hours, and the E-selectin expression level on the plasma membrane was analyzed by immunofluorescent staining using anti-E-selectin antibody. As a reference for the physiological level of E-selectin expression, the cells were also treated with TNF-α (10 ng/ml) for 5 hours. Elevated expression of E-selectin was detected predominantly on the cell membrane when treated with 500 ng/ml of doxycycline, and its expression level was increased in a doxycycline concentration dependent manner (Fig. 1A). In the absence of doxycycline, the baseline level of E-selectin expression was slightly higher than wild type cells, perhaps due to the leakiness of this inducible system. The level of E-selectin expression with a doxycycline concentration of 2000 ng/ml was equivalent to TNF-α treated cells, and thus, we used doxycycline at this concentration for subsequent experiments, unless otherwise specified. For the second-step selection of TAs that bind specifically to E-selectin, ES-Endo were pre-incubated with doxycycline and then with each of the 14 TAs (100 nM) selected in the first step for 20 minutes at 37°C. Fluorescent intensities associated with the cells were compared using fluorescent microscopy, and the data was evaluated for both binding and specificity. Interestingly, the relative binding affinities and specificities of the TA sequences were found to correlate with the energy of the structures predicted by MFOLD [34] (Table S1). Among the 14 TAs tested, TA-1 exhibited high doxycycline-dependent binding to ES-Endo with minimal binding to ES-Endo in the absence of doxycycline, whereas the rest of the TAs showed weak doxycycline dependent binding with high background (Table S1). The MFOLD prediction exhibited a single secondary structure for TA-1 (with an estimated free-energy of folding of −10.72 kcal/mol) containing two stable hairpin loops (Fig. 2B). In contrast MFOLD predicted four secondary structures for both TA-20 (dG = −7.98 to −7.44 kcal/mol) and TA-31 (dG = −8.64 to −7.94 kcal/mol) with comparable free energy values, and only a single stable hairpin loop was predicted in each of these structures (Fig S4). Interestingly, two highest binders (TA-1 and TA-20) share the ACT(T/C)C(T/A)C(T/C)TCAC sequence motif in the loop region of the hairpin stem-loop (Fig. S3), suggesting that this region might be involved in binding to E-selectin. Presumably the second hairpin loop in TA-1, but not in TA-20 contributes to its increased binding affinity and specificity. Overall, TA-1 showed the highest binding as well as specificity. Based on database search (NCBI BLAST), the TA-1 sequence (Fig. 2A) did not show any homology to existing genes. Together, we performed further studies using E-selectin thioaptamer TA-1 (ESTA-1).

To provide further concrete evidence that ESTA-1 binding to ES-Endo is E-selectin specific, we used three independent approaches. First, the ES-Endo cells were treated with increasing concentrations of doxycycline (up to 2000 ng/ml) for 5 hours to induce different levels of E-selectin expression, and they were then incubated with a fixed concentration of ESTA-1 (100 nM) for 20 minutes at 37°C. ESTA-1 was found to form a speckled binding pattern. The number and brightness of the speckles increased proportionally to the doxycycline concentration up to 2000 ng/ml (Fig. 1A), suggesting E-selectin specific ESTA-1 binding. Treatment of ES-Endo with TNF-α (10 ng/ml) yielded a similar binding pattern and fluorescent intensity (Fig. 1A). In the second approach, ES-Endo expressing E-selectin were first pre-treated with two different concentrations of E-selectin monoclonal antibody for 2 hours and then incubated with 100 nM of ESTA-1. Pre- and co-incubation of the cells with E-selectin monoclonal antibody caused a significant reduction of ESTA-1 binding as evidenced by the disappearance of the speckle pattern (Fig. 1B), suggesting that ESTA-1 and E-selectin antibody share the same epitope on E-selectin. Similar reduction in ESTA-1 binding was also observed by E-selectin antibody for TNF-α treated ES-Endo (data not shown). In contrast, normal IgG pre-treatment did not affect ESTA-1 binding to the cells (Fig. 1B). Thirdly, immunostaining was performed using a monoclonal antibody against E-selectin that does not compete with ESTA-1 binding to doxycycline induced ES-Endo (data not shown). This data demonstrated that ESTA-1 (red fluorescence) partially colocalized with E-selectin (green fluorescence) on the edge of the cells as seen in yellow merge, supporting ESTA-1 binding to E-selectin on the cell surface (Fig. S6). A possible explanation of a partial colocalization of ESTA-1 and E-selectin might be either ESTA-1 binding to other surface molecules on the endothelial cells or intracellular uptake following the initial cell surface binding since E-selectin undergoes internalization [34]. In conclusion, the two-step screening strategy employed here led to the identification of a TA sequence (ESTA-1) that binds specifically to E-selectin expressed on endothelial cells.

### ESTA-1 binding to tumor vasculature

**Figure 3:**
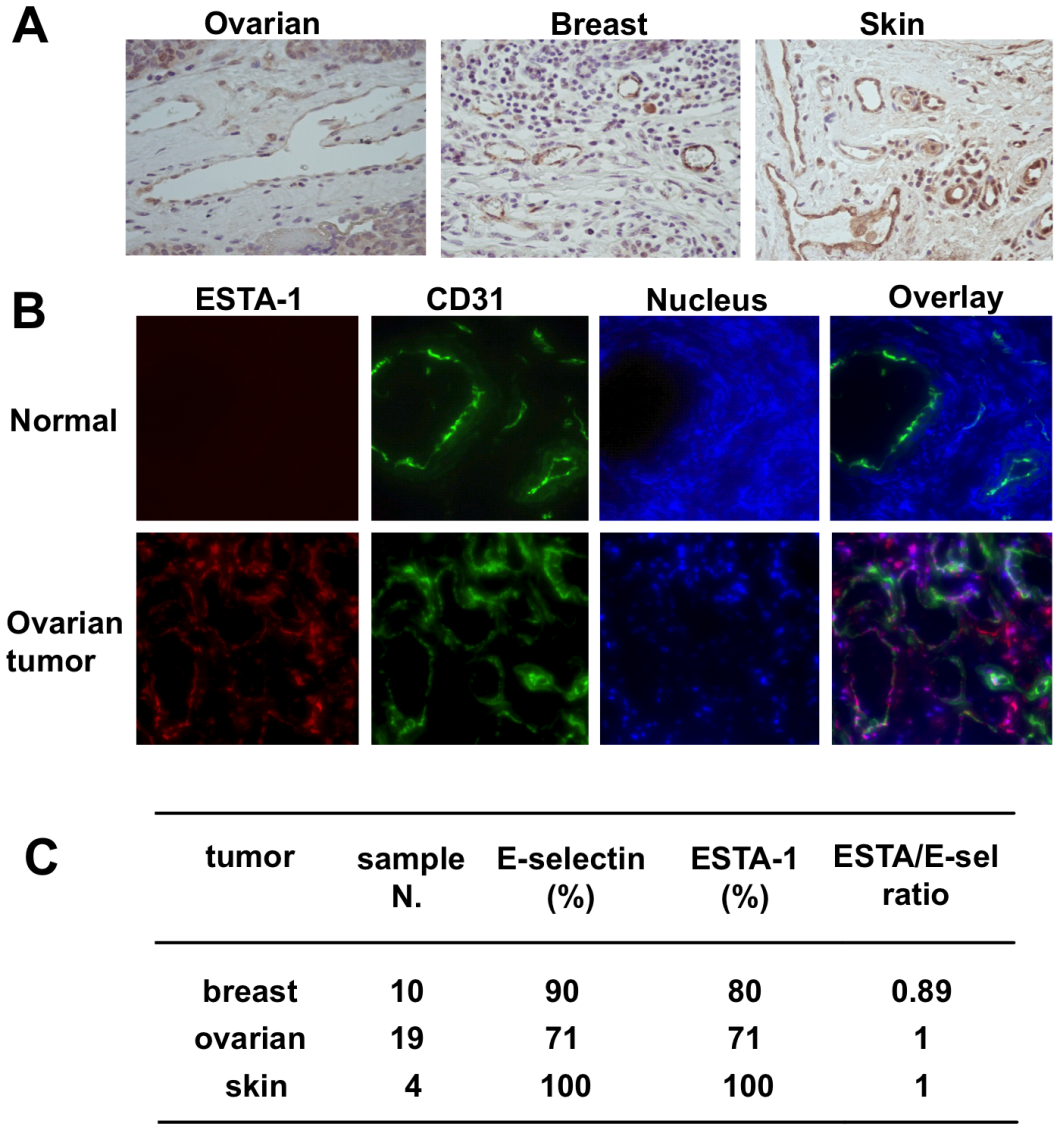
ESTA-1 binding to the tumor vasculature. Frozen sections derived from human ovarian carcinomas and normal ovaries were examined for E-selectin expression and ESTA-1 binding. (A) Immunohistochemical analysis for E-selectin expression on the vasculature of ovarian carcinoma. (B) ESTA-1 binding to tumor vasculature of ovarian carcinoma. At least five individual tumors were examined with five different fields per slide and representative sections were shown at the final magnification of x200. Green, CD31; Red, Cy3-labeled ESTA-1; Blue, Hoescht 33342. (C) Correlation of ESTA-1 binding to the tumor vasculature and E-selectin expression in human carcinomas derived from breast, ovary, and skin.

Next, we tested ESTA-1 binding to the tumor vasculature using histological sections derived from human carcinomas. First, immunohistochemical analysis was performed to evaluate the level of E-selectin expression on the tumor vasculature using paraffin sections derived from three types of carcinomas including breast, ovarian, and skin. Approximately 70-80% of tumors showed E-selectin expression on the vasculature (Fig. 3A and C). Unlike angiogenic factors such as integrins and vascular endothelial growth factor receptor, E-selectin expression was detected in both the existing mature vessels and the microvessels in the tumor (Fig. 3A). To test ESTA-1 binding to the tumor vasculature, the frozen sections were first incubated with a 50 nM solution of Cy3-labeled ESTA-1 (red fluorescence), then immunostained with CD31 (green fluorescence). Intense ESTA-1 binding was observed on the vessels in ovarian carcinomas as evidenced by the co-localization with CD31 (Fig. 3B). In contrast, ESTA-1 binding was not observed in the vessels in the normal counterpart. Similarly, ESTA-1 bound to the tumor associated vessels in breast (80%) and skin (100%) carcinomas (Fig. 3C). Overall, ESTA-1 binding to the tumor associated vessels was highly correlated with E-selectin expression as indicated by ESTA/E-sel ratios for breast (ratio = 0.89), ovarian (ratio = 1) and skin (ratio = 1) carcinomas (Fig. 3C). As opposed to the binding to the tumor associated vessels, ESTA-1 binding was almost absent in the normal human tissues, including the adrenal, brain, temporal lobe, breast, cervix, heart, kidney, liver, lung, pancreas, placenta, salivary gland, skeletal muscle, small intestine, spleen, stomach, thyroid, and uterus, with the exception of minor binding to the vessels of the skin (data not shown), where E-selectin is constitutively expressed [35].

**Figure 4:**
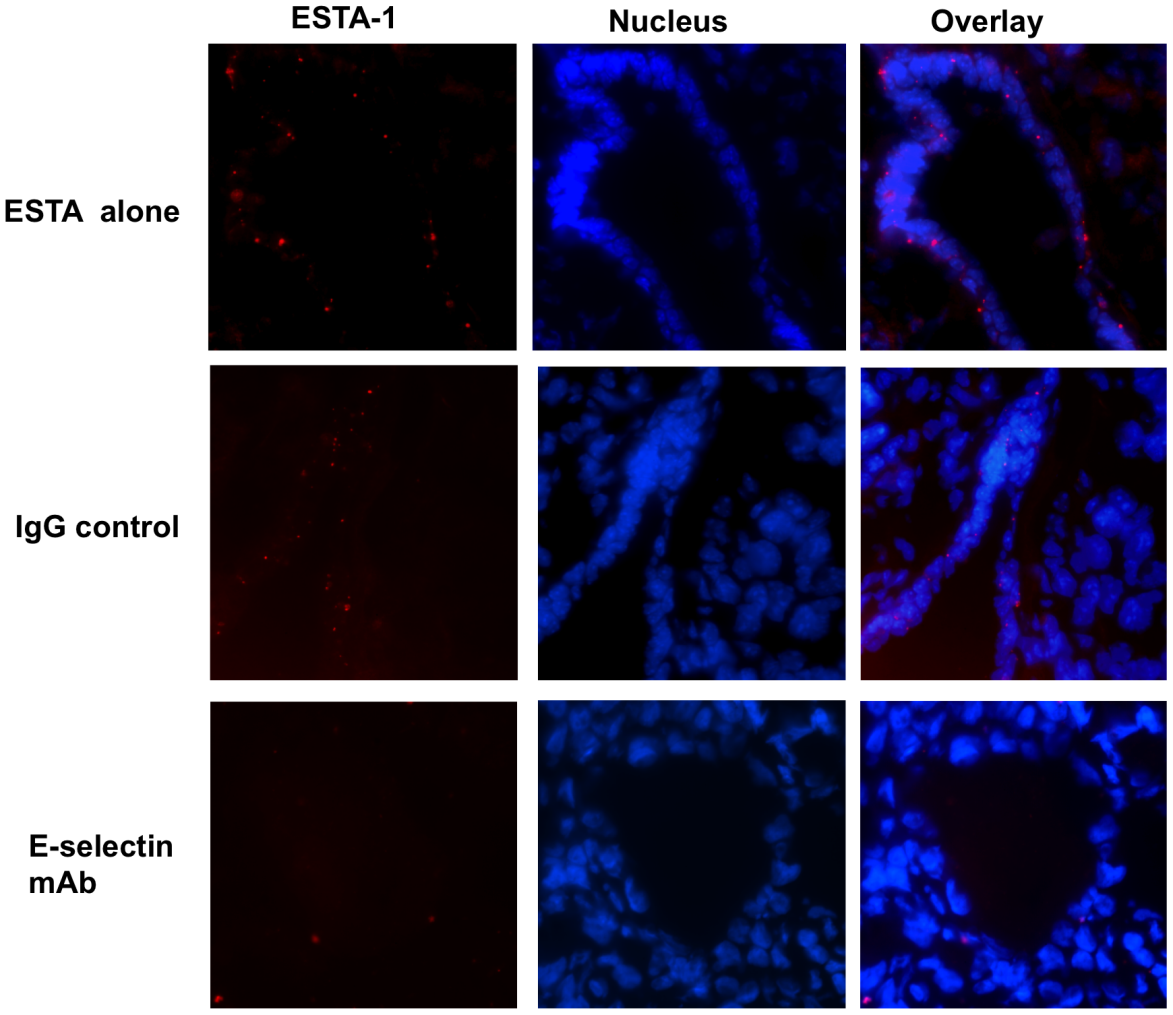
*In vivo* distribution of ESTA-1 in mouse bearing xenograft tumor derived from breast cancer 4T1 cells. Frozen sections derived from 4T1 xenograft model were examined for E-selectin expression and ESTA-1 binding. ESTA-1 (10 µg/100 µl saline) was injected to mice (n = 3) via tail vein and organs, including liver, kidney, lung, heart, spleen, and tumor, were harvested 5 hours after the injection. Frozen sections (5 µm) were prepared to assess distribution of ESTA-1. Red, Cy3-labeled ESTA-1; blue, Hoechst 33342.

Next we tested ESTA-1 binding to E-selectin on tumor-associated vasculature *in vivo.* We used a 4T1 breast tumor mouse model in which high E-selectin expression was observed on the endothelial cells of the tumor-associated vasculature (Supplemental Fig. S5). Intravenous administration of ESTA-1 into mice bearing 4T1 tumor resulted in accumulation of ESTA-1 to the endothelial cells of the tumor vasculature as evidenced by the speckled red pattern on the tumor associated vasculature (Fig. 4). No obvious binding of ESTA-1 to the vasculature of other organs (liver, spleen, kidney, lung, and heart) was detected (data not shown). To confirm E-selectin specific binding of ESTA-1 *in vivo*, an E-selectin antibody was injected intravenously prior to ESTA-1 injection into mice bearing breast tumor xenograft. E-selectin antibody pre-injection resulted in a significant reduction in ESTA-1 binding to tumor vasculature as compared to untreated mice, whereas pre-injection of control IgG did not cause any changes (Fig. 4), suggesting that ESTA-1 binds to E-selectin on the tumor vasculature.

### Evaluation of binding affinity of ESTA-1 to E-selectin

**Figure 5:**
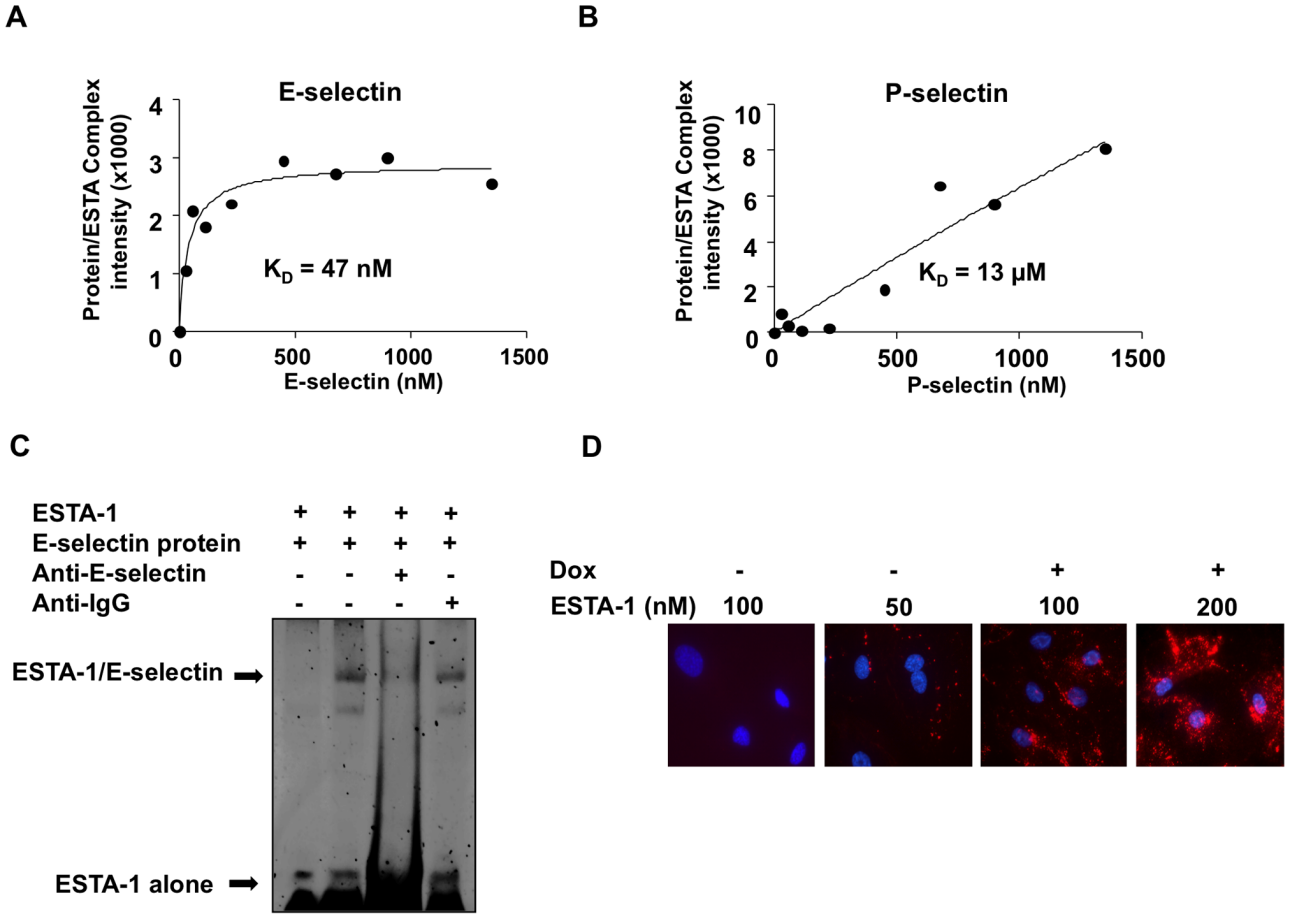
Evaluation of binding affinity of ESTA-1 to E-selectin. ESTA-1 (4.6 pmoles) and recombinant human E-selectin protein (up to 19 pmoles) were incubated and subjected to electrophoresis at 4 °C. The gels were stained with SYBR Gold nucleic acid stain and densitometric analysis of the unbound ESTA-1 was plotted. (A) E-selectin recombinant protein. (B) P-selectin recombinant protein. (C) Competition of ESTA-1 binding to E-selectin protein. Human recombinant protein was pre-incubated with E-selectin antibody (3 µg) for 30 min prior to addition of ESTA-1 (4.6 pmoles). EMSA was carried out and gel was stained with SYBR Gold stain to analyze ESTA-1 binding. (D) ESTA-1 concentration dependent binding to ES-Endo. ES-Endo were incubated with doxycycline (1000 ng/ml) for 5 hours and then with indicated concentrations of Cy3-labeled ESTA-1 for 20 minutes. ESTA-1 binding was analyzed by fluorescent imaging. Red, Cy3-labeled ESTA-1; blue, Hoechst 33342.

We next evaluated the binding affinity of ESTA-1 to all selectins using electrophoretic mobility shift assay (EMSA). To determine the binding constant, fixed amounts of ESTA-1 was mixed with increasing amounts of recombinant protein (E-, P-, and L-selectin). Incubation of recombinant E-selectin protein and ESTA-1 resulted in the formation of a DNA/protein complex in equilibrium with unbound states. An increment in ESTA-1/E-selectin complexes was observed with increasing recombinant E-selectin added to the reaction, accompanied by a corresponding decrease in the free (unbound) ESTA-1 (data not shown). As expected, the amounts of ESTA-1/E-selectin complexes reached saturation at a molar ratio of 1∶1, when both of the binding molecules are at a concentration of 500 nM. Based on the densitometric analysis, the binding constant calculated for the ESTA-1 binding to E-selectin was 47 nM (Fig. 5A). The binding of ESTA-1 to P-selectin showed significantly lower affinity (estimated K_D_ = 13 µM), suggesting a very weak interaction at the concentration range measured (Fig. 5B). ESTA-1 binding to L-selectin was not detectable under the same conditions. To validate ESTA-1 binding to E-selectin, EMSA was performed by pre-incubating the human E-selectin recombinant protein with E-selectin antibody (H18/7) prior to addition of ESTA-1 to the reaction mixture. The addition of E-selectin antibody (H18/7) resulted in the disappearance of the band corresponding to the ESTA-1/E-selectin complex (Fig. 5C). This data implies that ESTA-1 not only binds to E-selectin, but also may share the same binding site with E-selectin monoclonal antibody used for this experiment. To further confirm the binding affinity of ESTA-1 under biological conditions, different concentrations of ESTA-1 (50–200 nM) were incubated with ES-Endo induced with doxycycline. ESTA-1 binding to the cells was detectable at 50 nM and increased in a dose dependent manner (Fig. 5D). Together, these data support a nanomolar affinity of ESTA-1 binding to E-selectin on the endothelial cells.

### ESTA-1 inhibits HL-60 cell adhesion to endothelial cells

**Figure 6:**
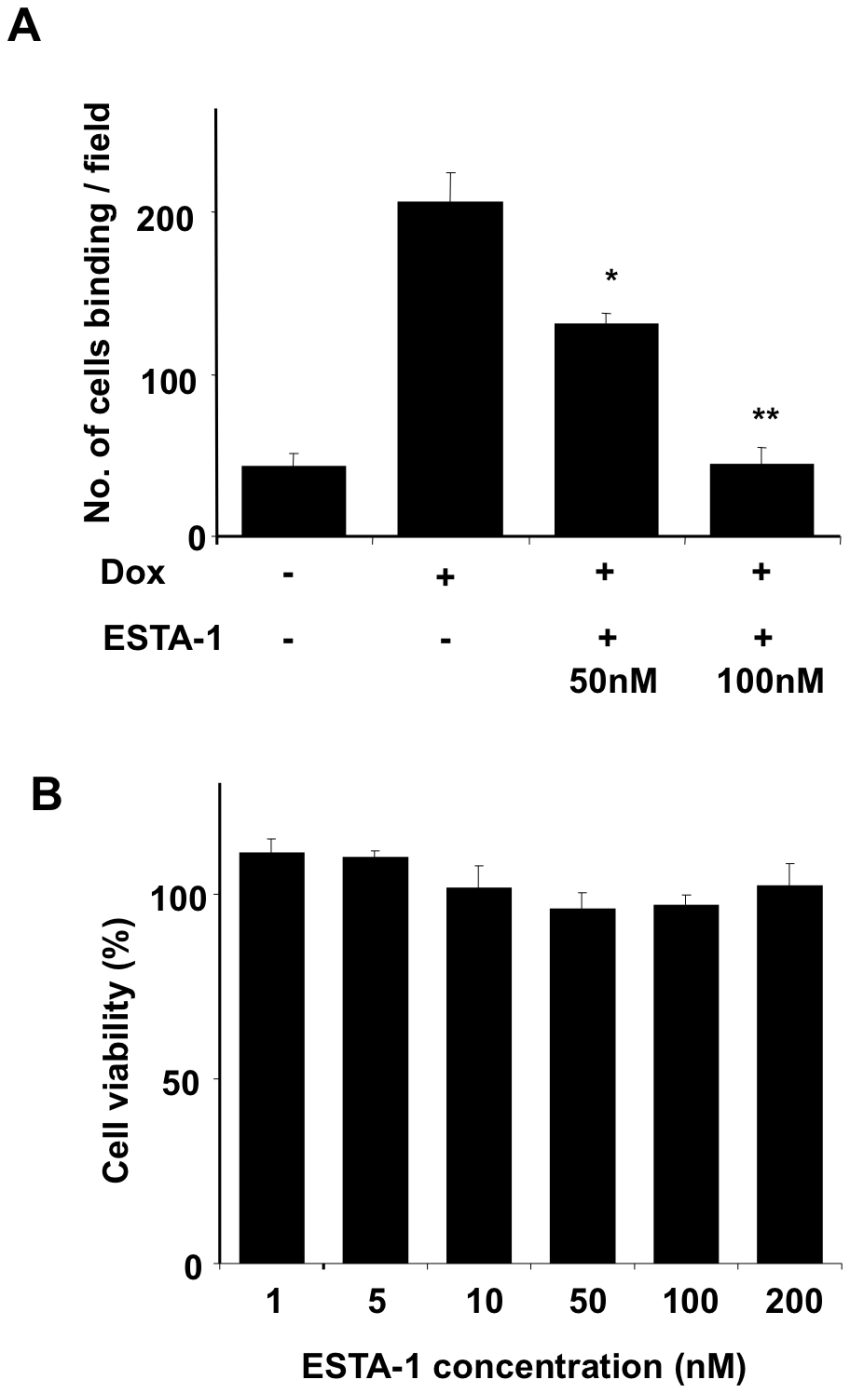
Effect of ESTA-1 binding on cell adhesion and cell viability. ES-Endo were incubated with doxycycline (1000 ng/ml) for 5 hours followed by two concentrations of ESTA-1 (50 and 100 nM) for 30 minutes. sLe^x^ positive HL-60 cells were added to each well and incubated at 4 °C for 30 minutes and cell adhesion was analyzed under 100x final magnification (A). Error bars, mean ± SEM; *,P<0.05, **, P<0.01 vs. Dox+/ESTA-, Student's t test. ES-Endo were induced with doxycycline (2000 ng/ml) for 5 hours and incubated with culture media containing ESTA-1 at indicated concentrations for 48 hours (B). The cells were washed and incubated with MTT for 4 hours, and the absorbance at 570 nm was measured. The data was normalized by untreated cells (without ESTA-1) as 100%. Experiments were repeated three times.

On the basis of specific binding of ESTA-1 to E-selectin, we next tested ESTA-1-mediated inhibition of leukocyte adhesion to endothelial cells. For this study, we tested a human promyelomonocytic cell line (HL-60) that expresses sLex, a natural ligand for E-selectin. HL-60 cell adhesion to ES-Endo increased by 5-fold when E-selectin expression was induced by doxycycline (Fig. 6A). E-selectin expressing ES-Endo were pre-incubated with indicated concentrations of ESTA-1 for 20 minutes and then the adhesion of these cells to the ES-Endo was compared. Pretreatment of the E-selectin expressing ES-Endo with ESTA-1 inhibited HL-60 adhesion by 80% at 100 nM ESTA-1 (Fig. 6A) (p<0.01). The IC_50_ for the inhibition of this interaction was approximately 63 nM. These data indicate that the ESTA-1 interaction to E-selectin occurs through the sLe^x^-binding site, further highlighting the potential therapeutic utility of ESTA-1 as an antagonist of E-selectin mediated adhesion. Lastly, we tested the cytotoxicity associated with ESTA-1 treatment in ES-Endo. ES-Endo were first incubated with doxycycline for 5 hours and then with increasing concentration of ESTA-1 (up to 200 nM) for 48 hours. MTT assay was performed to test cell viability. Incubation with up to 200 nM of ESTA-1 for 48 hours did not cause any visible morphological changes or a reduction of cell viability in ES-Endo cells (Fig. 6B).

## Discussion

In this study, we have identified a thioaptamer ligand against E-selection (ESTA-1) with a 47 nM binding affinity to E-selectin, and importantly, with minimum cross reactivity to P- or L- selectin. We also demonstrated that ESTA-1 bound efficiently to E-selectin expressing endothelium of human and mouse carcinomas. Furthermore, ESTA-1 effectively inhibited the adhesion of sLex positive HL-60 cells on E-selectin expressing endothelial cells. These data accentuate the versatile biomedical applications of ESTA-1 for E-selectin targeted therapies and imaging of inflamed tumor vasculature.

Inflamed vascular endothelium has been recognized as an attractive site for targeted delivery of therapeutic and imaging agents because of significant differences in the expression of surface receptor proteins between normal and inflamed endothelium. E-selectin expression on the vasculature is transcriptionally induced in the presence of inflammatory stimuli, and subsequently, E-selectin expression is commonly observed in pathological inflammation, including cancer [7]. Additionally, following membrane sorting upon cytokine stimuli, E-selectin undergoes a recycle phase, rapid internalization to endosomes and subsequent partial lysosomal degradation. These characteristics, inflammation-dependent expression and internalization, make E-selectin an attractive target for intracellular delivery of therapeutics to inflamed vasculature. Many different types of E-selectin affinity ligands, including a humanized monoclonal antibody, peptide and carbohydrate mimetics, have been developed for biomedical applications [19], [20]. However, low affinity (µM range K_D_) and unfavorable pharmacokinetics of these ligands limits their potential application for targeted delivery, thus highlighting the need for a high affinity E-selectin ligand with improved pharmacokinetics and serum stability.

Aptamers are an emerging class of ligands that have several advantages over antibodies or peptide-based ligands; lack of immunogenicity and toxicity [36]. Since native oligonucleotides are susceptible for hydrolysis by nucleases, stabilization of the phosphate backbone has been one of the major focuses for biomedical applications. Although modification with neutral groups such as methyl phosphonate [37] and phosphoramidate [38] have shown increased resistance to nucleases, these analogs showed lower binding affinity than the unmodified oligonucleotides. In contrast, sulfur substitution of the phosphoryl oxygens, either one (monothio) or two (dithio) of the non-bridging oxygen atoms, exhibit enhanced affinity to protein as well as resistance against nucleases in both cellular and plasma environments [39]. Based on molecular dynamics and theoretical calculations, we and others have suggested that increased affinity of thioaptamer may be attributed to the decreased interaction of solvated cations with the sulfur atoms, which act as softer Lewis bases on the polyanionic backbone [40]. Our data demonstrated that monothiophosphate substitution in ESTA-1 resulted in high affinity binding to E-selectin (47 nM) and minimum cross reactivity (Fig. 5). This is a significant improvement of 2,000-40,000 times higher affinity as compared to the natural ligand sLe^x^ (K_D_  = 100–2000 µM) (13). Moreover, dithio-substituted DNA has been shown to increase the binding affinity by 100–600 times higher than the non-thioated or monothio analogue [30]. This suggests that dithio-substitution can lead to further development of higher affinity of ligands against E-selectin.

For the identification of E-selectin specific thioaptamer, we utilized a two-step selection strategy. The first step involving 10 iterative cycles of combinatorial library screening using extracellular domain of human E-selectin recombinant protein led to the identification of 14 TA sequence families. In the second step cell based screening, to our surprise, only one of the 14 selected TAs exhibited highly doxycycline-dependent binding to endothelial cells expressing E-selectin, despite the initial screening using human recombinant E-selectin protein isolated from the mammalian system. These data indicated that *in vitro* selection with pure biochemical entities (e.g., recombinant protein) in solution may not readily translate into effective ligand binding in a complex biological environment, particularly if the target protein is significantly post-translationally modified. In fact, E-selectin is a glycoprotein that is matured from 107 kDa to 115 kDa through sequential post-translational modification with carbohydrates [8]. Screening of aptamer combinatorial libraries often utilizes either full-length or fragments of recombinant proteins [41], [42], [43], [44]. However, the structural differences that result from the lack of post-translational modifications and possible misfolding of these recombinant proteins may preclude the identification of aptamers that would maintain their binding in a physiological environment. Thus, the integration of biologically relevant conditions into the screening process is essential for the identification of aptamers that could show relevant biological activity. In fact, various screening strategies that have incorporated cellular screening, rather than single molecule based screening with recombinant proteins, resulted in the identification of novel aptamers for specific target molecules or even target cell type [45].

For the second step screening, we employed an E-selectin Tet-on inducible system that allows for highly controllable and selective E-selectin expression for identification of E-selectin specific binders. The most widely accepted approach to induce E-selectin expression relies on cytokine stimulation such as IL-1β and TNF-α that mimics gross phenotypic changes on endothelial cell surface (i.e., induction of surface receptors including E-selectin, P-selectin, and cell adhesion molecules); however, such approaches are unlikely to rule out the involvement of other cell surface molecules. In fact most of the carbohydrate mimetics and ligands identified against E-selectin have shown considerable cross reactivity against L- and P- selectin due to their structural similarities [23], thereby limiting the use of such for targeted delivery due to the possible off-targeting effects. Overall, the two-step screening strategy with integration of cell based binding using Tet-on inducible ES-Endo cells allowed us to identify a novel E-selectin thioaptamer (ESTA-1) that specifically binds to E-selectin expressing on the endothelial cell surface (Fig. 1A). The major pitfall of the cell-based approach might be a species difference between origin of the cells for screening and experimental animal model for in vivo validation of binding. The use of human cell or cell lines for ligand screening can be well rationalized for eventual biomedical application. However, ligand binding specificity and affinity to the target molecule may not be well validated in experimental animal models, disabling pre-clinical study. Therefore, we tested ESTA-1 binding to E-selectin expressing vessels in both human carcinoma pathology samples and 4T1 breast cancer xenograft animal model. Despite species difference between human and mouse, intravenously administered ESTA-1 was found predominantly on the vessel surface of the tumor-associated vasculature in mouse, (Fig. 4) possibly due to the sequence and structural homology between human and mouse E-selectin [46]. This species independent binding of ESTA-1 to E-selectin, at least in human and mouse, enables thorough characterization of this ligand in a variety of mouse disease models for possible biomedical application.

To determine the specificity of the ESTA-1 binding to E-selectin, we carried out multiple approaches. First in vitro, pre-incubation of an E-selectin monoclonal antibody with E-selectin protein resulted in a disappearance of the band corresponding to the ESTA-1/E-selectin complex in EMSA (Fig. 5C). In *ex vivo*, pre-incubation of the E-selectin antibody with doxycycline induced ES-Endo followed by addition of ESTA-1 showed reduction of ESTA-1 binding in an antibody dose dependent manner (Fig. 1B). Finally *in vivo*, pre-injection of E-selectin antibody prior to ESTA-1 injection lead to a reduction in ESTA-1 binding to tumor vasculature *in vivo* (Fig. 4). Aside from antibody competition, immunostaining of the cells treated with ESTA-1 with E-selectin antibody that does not compete with the same binding site demonstrated partial colocalization of ESTA-1 with E-selectin (Fig. S6). Collectively, these results confirm the binding of ESTA-1 to E-selectin on the endothelial cells both *in vitro* and *in vivo.*

Although we focused on cancer in this study, versatile application beyond cancer can be considered on the basis of highly selective binding of ESTA-1 to E-selectin as well as a broad array of diseases that are associated with pathological inflammation. Moreover, recent development of nanotechnology based drug delivery and imaging would greatly benefit from selective, high affinity, and less immunogenic ligands [47], [48]. In fact, nanoparticles conjugated with E-selectin ligands, both peptide and monoclonal antibodies against E-selectin have shown efficient imaging of inflammation in tumor and rheumatoid arthritis in both an experimental animal model and humans [49], [50], [51]. Since thioaptamers are virtually non-immunogenic and highly nuclease resistant, the use of such a stable ligand for active targeting is likely to enhance delivery efficacy of therapeutics to the target sites. Together, to the best of our knowledge, this is the first report of an aptamer ligand to demonstrate nanomolar affinity binding to E-selectin, and presents a potential opportunity for broad application of ESTA-1 for inflamed vessel targeting *via* E-selectin.

## Supporting Information

Table S1 Comparison of TA binding to E-selectin expressing endothelial cells. The table shows the calculated lowest free energy for each 14 TA sequences, their relative binding, and relative specificities to E-selectin expressing cells. The relative binding affinity was determined by the amount of fluorescence detected per field of view (final magnification 60x) in the cell based binding assay and the relative specificity was defined by the degree of doxycycline dose dependent effect on TA binding. + indicates the binding specificity.
(0.14 MB TIF)

Figure S1 ClustalW alignment of the selected sequences after round 10. After the 10th round of selection, 35 clones were selected and their sequences were identified. The PCR primer regions in the sequences are underlined.
(0.67 MB TIF)

Figure S2 Cladogram of the selected sequences after round 10. The sequences from 10th round of selection were aligned by ClustalW. Based on the Phylogeny of the sequences they were grouped into 14 different families. A single sequence from each family was taken for the 2nd step cell based screening.
(0.21 MB TIF)

Figure S3 Common sequence motifs among 14 TA candidates (A) The 14 sequences belonging to each family from the cladogram are aligned by ClustalW program. (B) Common sequence motifs identified among the 14 sequences.
(0.47 MB TIF)

Figure S4 MFOLD predicted secondary structures of TA-20 and TA-31. The secondary structures of the selected sequences were obtained using the MFOLD program (at ambient temperature with ionic conditions of 150 mM Na+ and 5 mM Mg2+). TA-20 and TA 31 show 4 secondary structures with free energy values ranging between −7.98 to −7.44 kcal/mol and −8.64 to −7.94 kcal/mol respectively. Predicted structures of both TA-20 and TA-31 show a single stable stem loop in their structures.
(0.49 MB TIF)

Figure S5 Immunohistochemical analysis for E-selectin expression on the vasculature of 4T1 tumor. Frozen sections (5 µm) derived from 4T1 xenograft model were examined for E-selectin expression.
(1.31 MB TIF)

Figure S6 Colocalization of E-selectin expression and ESTA-1 binding to ES-Endo. ES-Endo cells were treated with doxycycline (2000 ng/ml) and analyzed for ESTA-1 binding and E-selectin expression using immunofluoroscence. Blue, Hoescht 33342; Red, Cy3-labeled ESTA-1; Green, E-selectin.
(0.60 MB TIF)
