# Supplementary figures and images for: Identification of Thioaptamer Ligand against E-Selectin: Potential Application for Inflamed Vasculature Targeting
Source: PLoS One. 2010 Sep 30;5(9):e13050. doi: 10.1371/journal.pone.0013050 (PMC2948018; doi:10.1371/journal.pone.0013050)

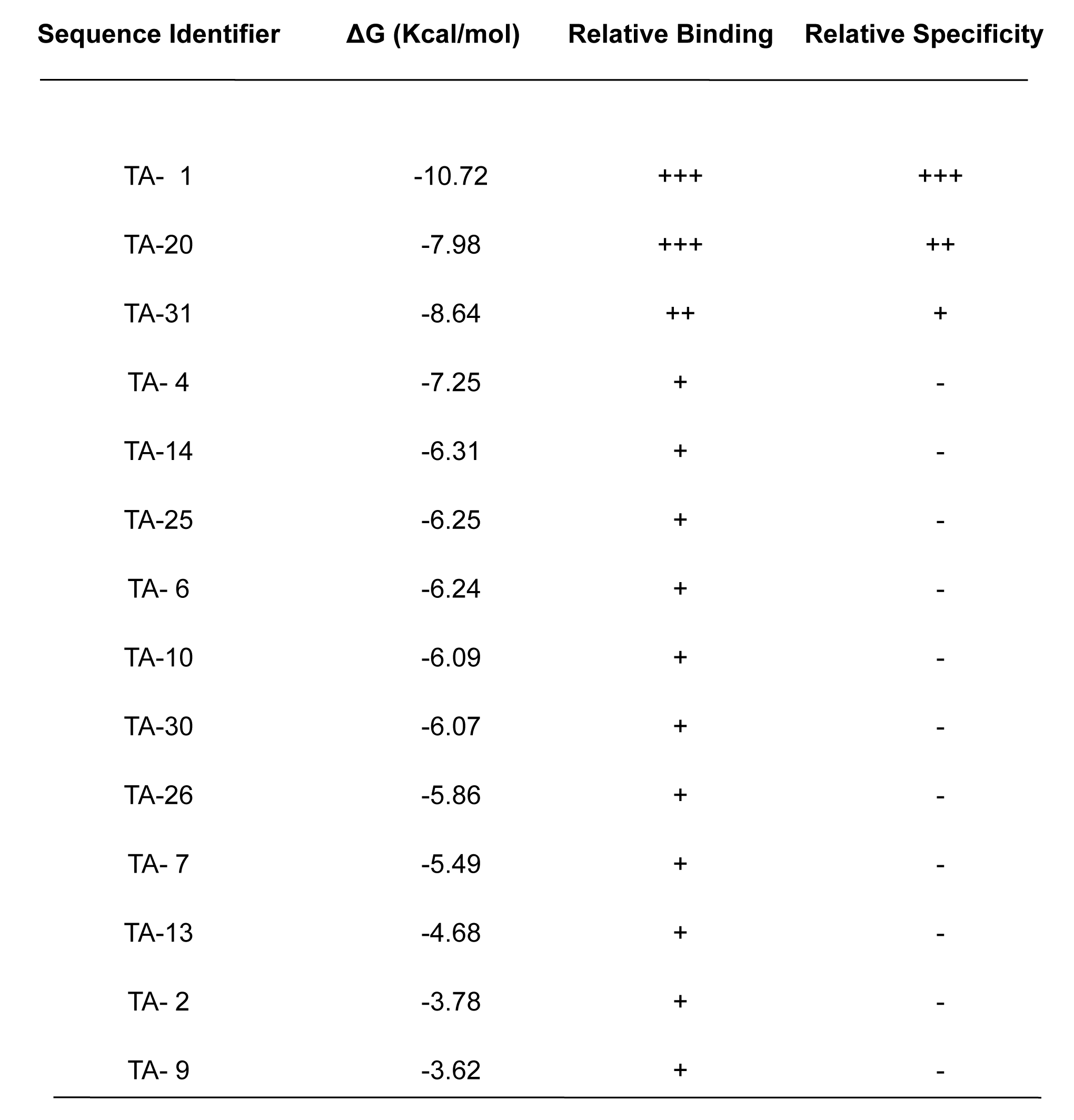

Supplement: Table S1 — Comparison of TA binding to E-selectin expressing endothelial cells. The table shows the calculated lowest free energy for each 14 TA sequences, their relative binding, and relative specificities to E-selectin expressing cells. The relative binding affinity was determined by the amount of fluorescence detected per field of view (final magnification 60x) in the cell based binding assay and the relative specificity was defined by the degree of doxycycline dose dependent effect on TA binding. + indicates the binding specificity. (0.14 MB TIF) [file pone.0013050.s001.tif]

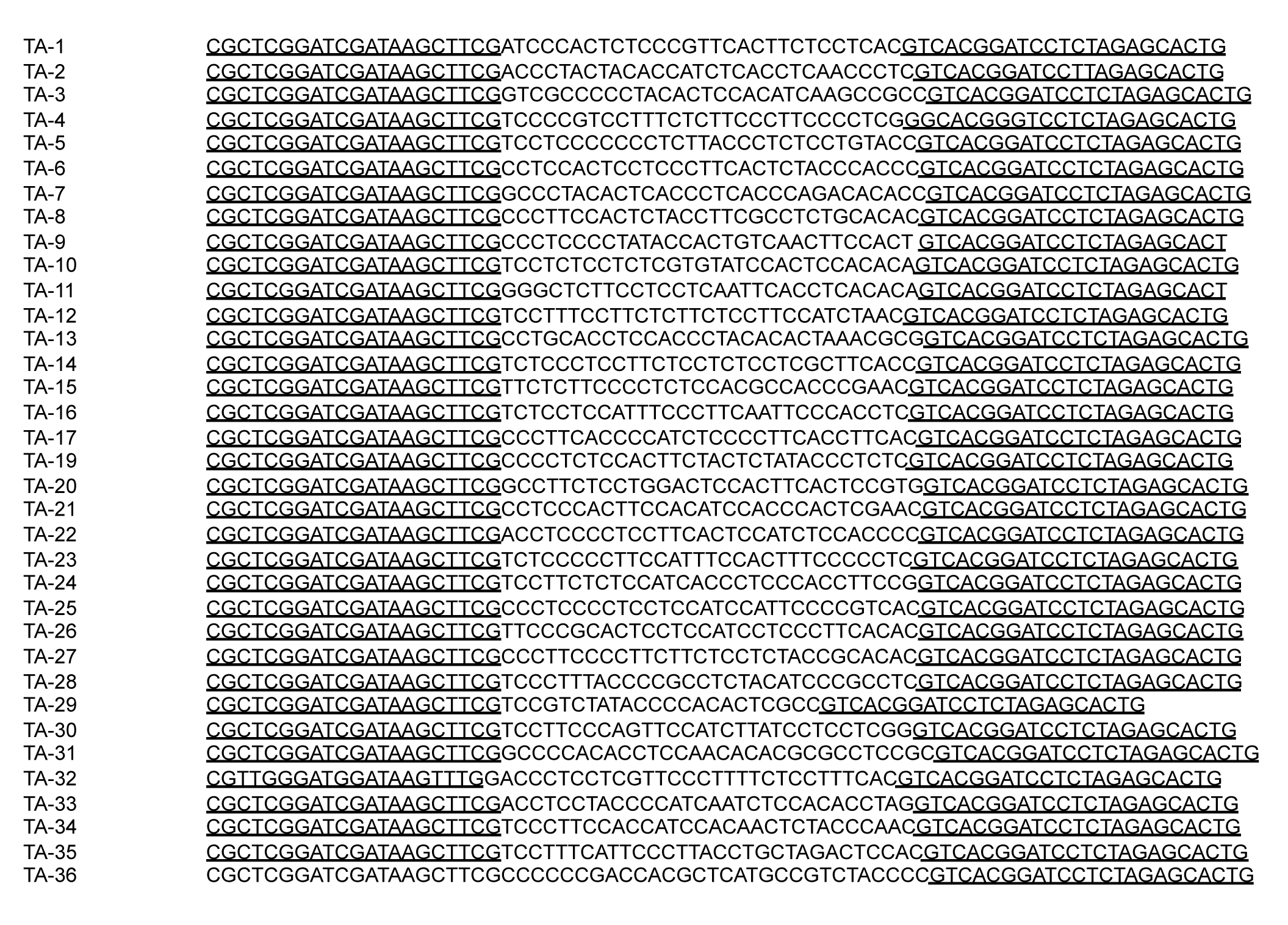

Supplement: Figure S1 — ClustalW alignment of the selected sequences after round 10. After the 10th round of selection, 35 clones were selected and their sequences were identified. The PCR primer regions in the sequences are underlined. (0.67 MB TIF) [file pone.0013050.s002.tif]

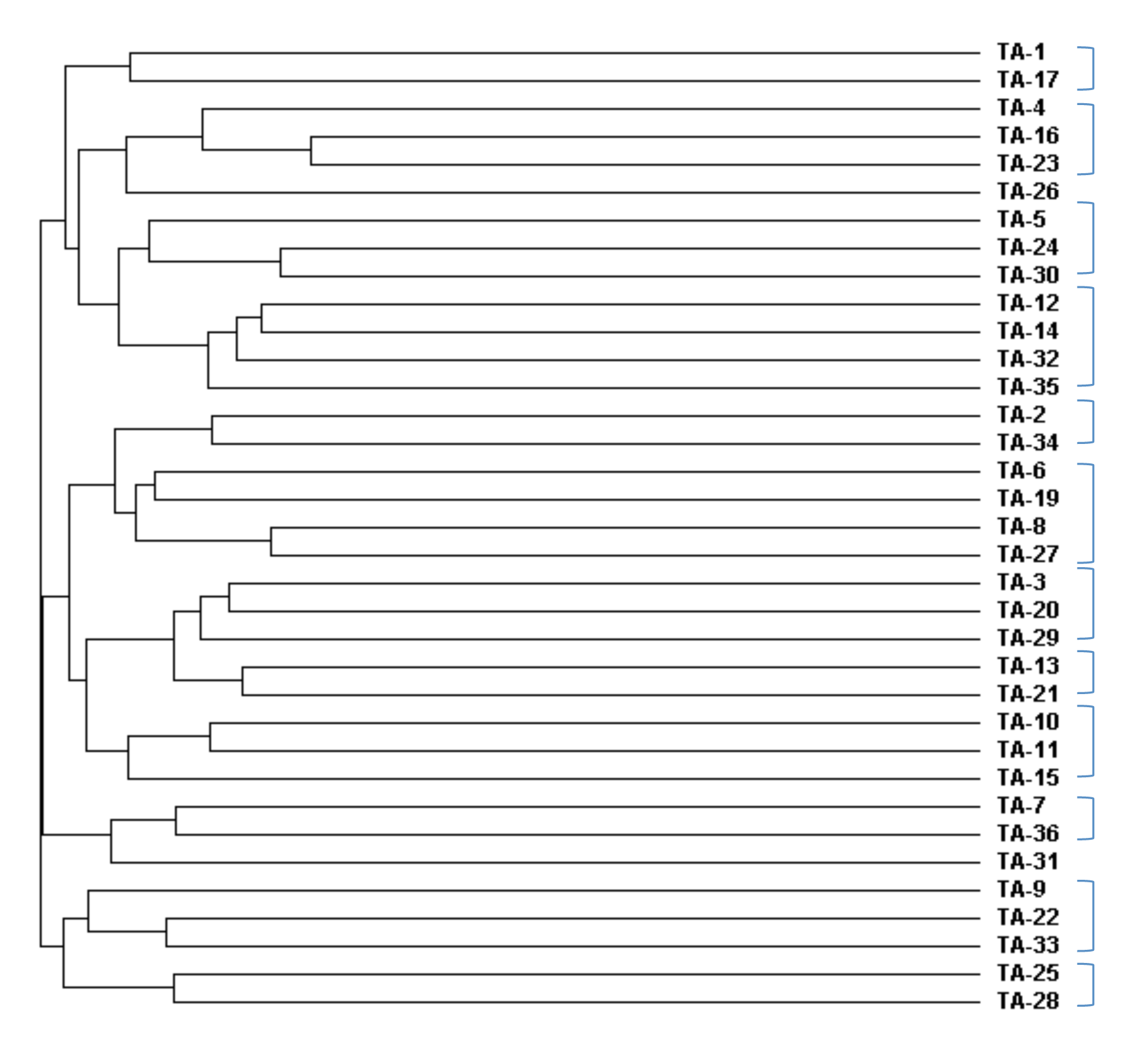

Supplement: Figure S2 — Cladogram of the selected sequences after round 10. The sequences from 10th round of selection were aligned by ClustalW. Based on the Phylogeny of the sequences they were grouped into 14 different families. A single sequence from each family was taken for the 2nd step cell based screening. (0.21 MB TIF) [file pone.0013050.s003.tif]

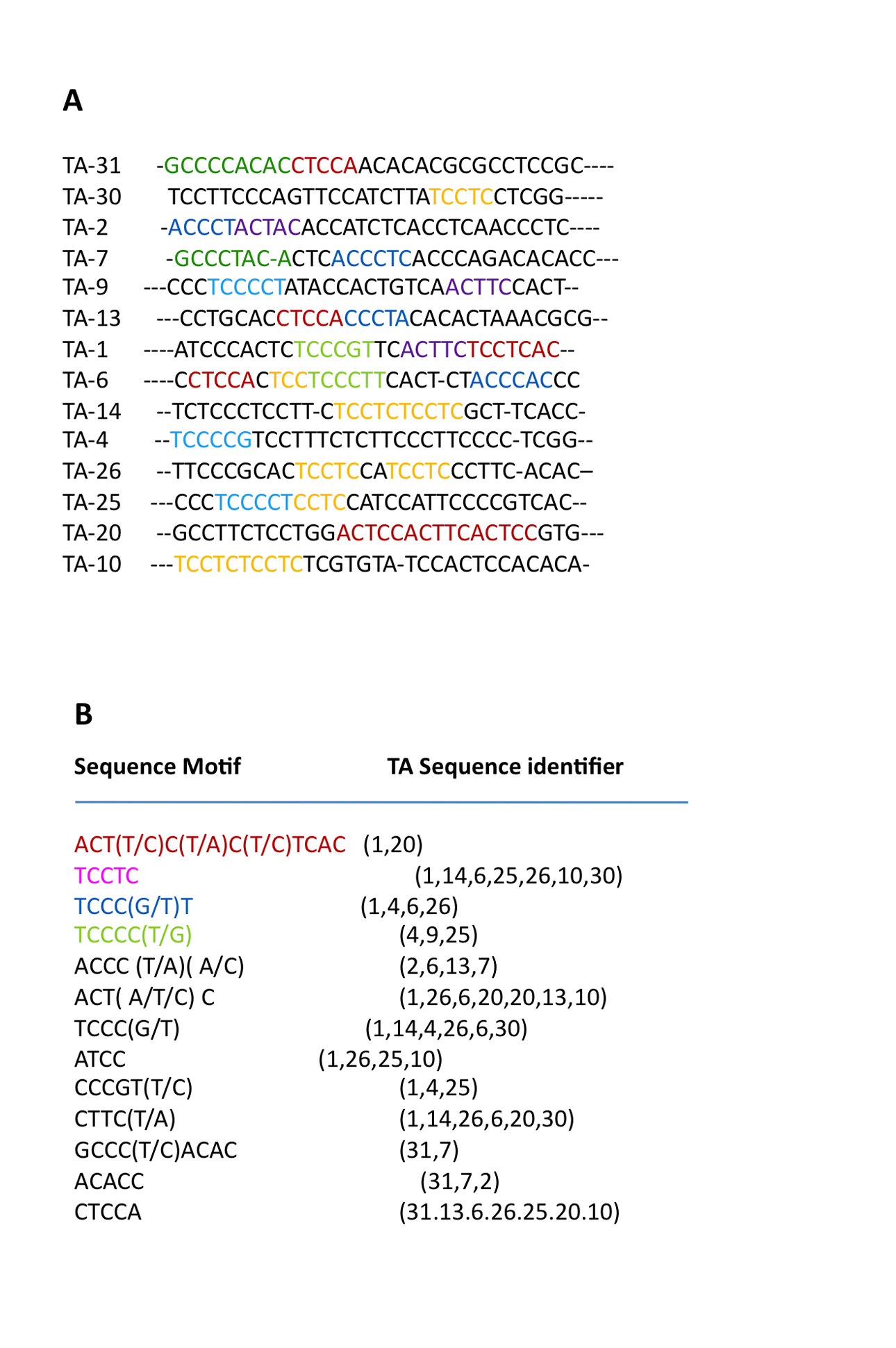

Supplement: Figure S3 — Common sequence motifs among 14 TA candidates (A) The 14 sequences belonging to each family from the cladogram are aligned by ClustalW program. (B) Common sequence motifs identified among the 14 sequences. (0.47 MB TIF) [file pone.0013050.s004.tif]

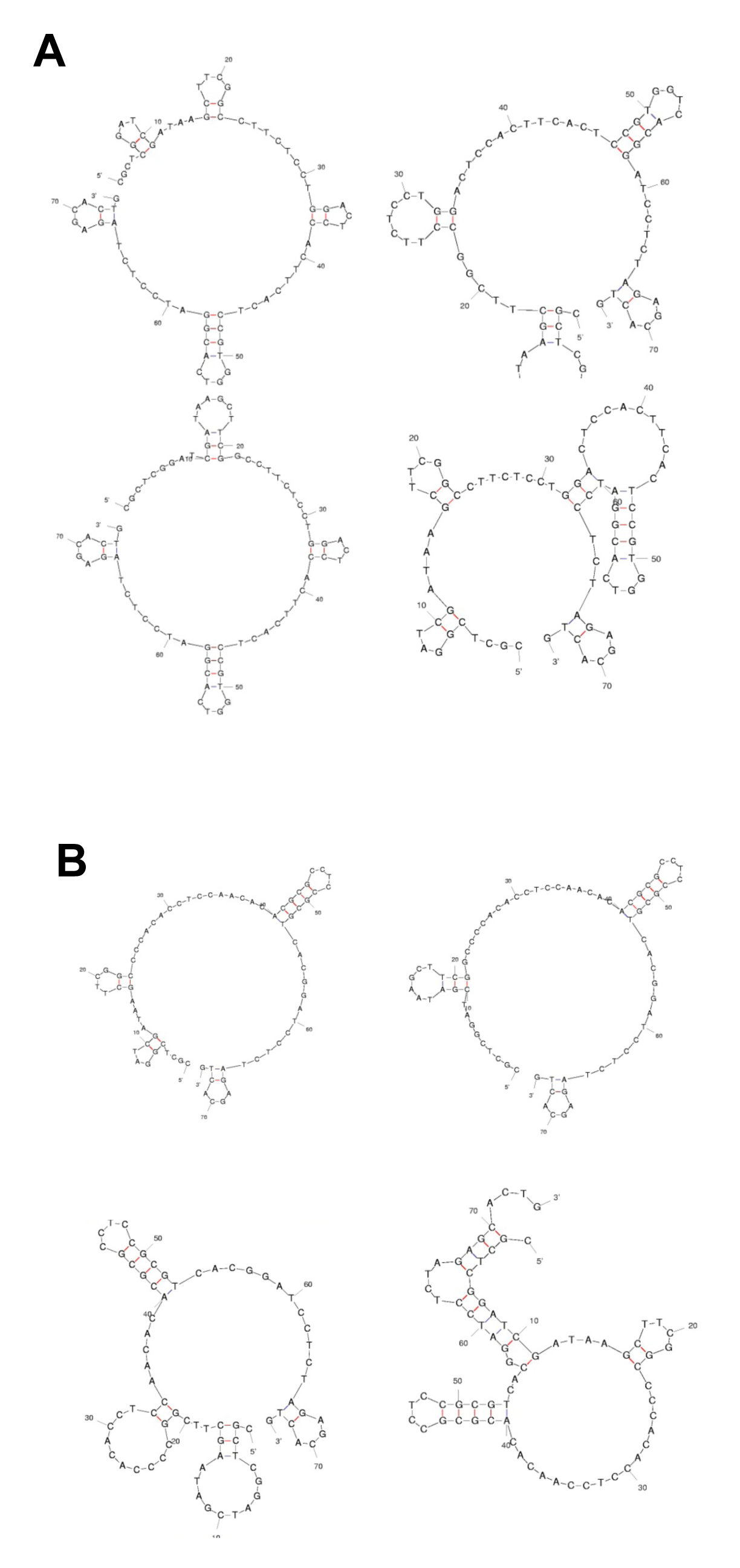

Supplement: Figure S4 — MFOLD predicted secondary structures of TA-20 and TA-31. The secondary structures of the selected sequences were obtained using the MFOLD program (at ambient temperature with ionic conditions of 150 mM Na+ and 5 mM Mg2+). TA-20 and TA 31 show 4 secondary structures with free energy values ranging between −7.98 to −7.44 kcal/mol and −8.64 to −7.94 kcal/mol respectively. Predicted structures of both TA-20 and TA-31 show a single stable stem loop in their structures. (0.49 MB TIF) [file pone.0013050.s005.tif]

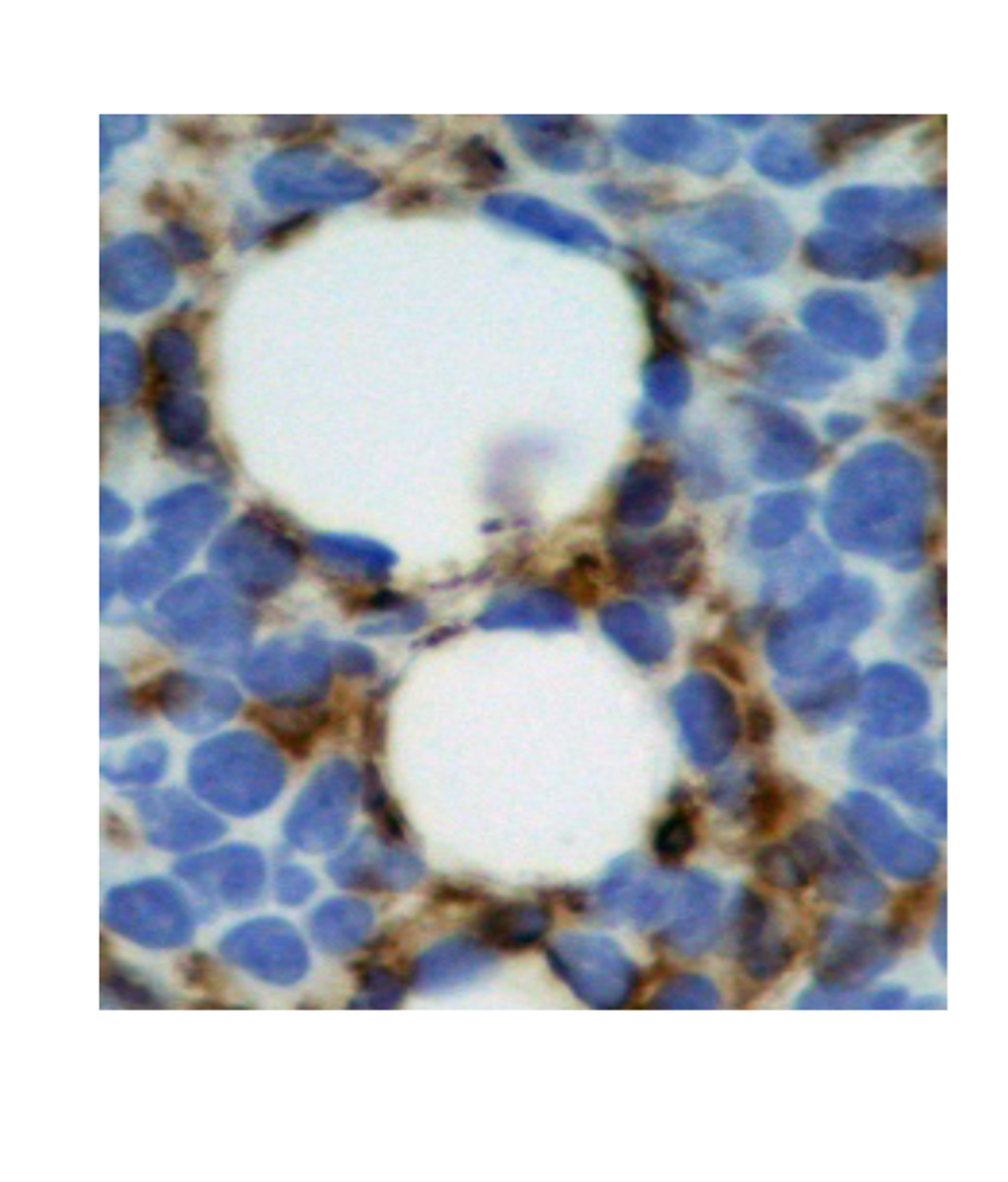

Supplement: Figure S5 — Immunohistochemical analysis for E-selectin expression on the vasculature of 4T1 tumor. Frozen sections (5 µm) derived from 4T1 xenograft model were examined for E-selectin expression. (1.31 MB TIF) [file pone.0013050.s006.tif]

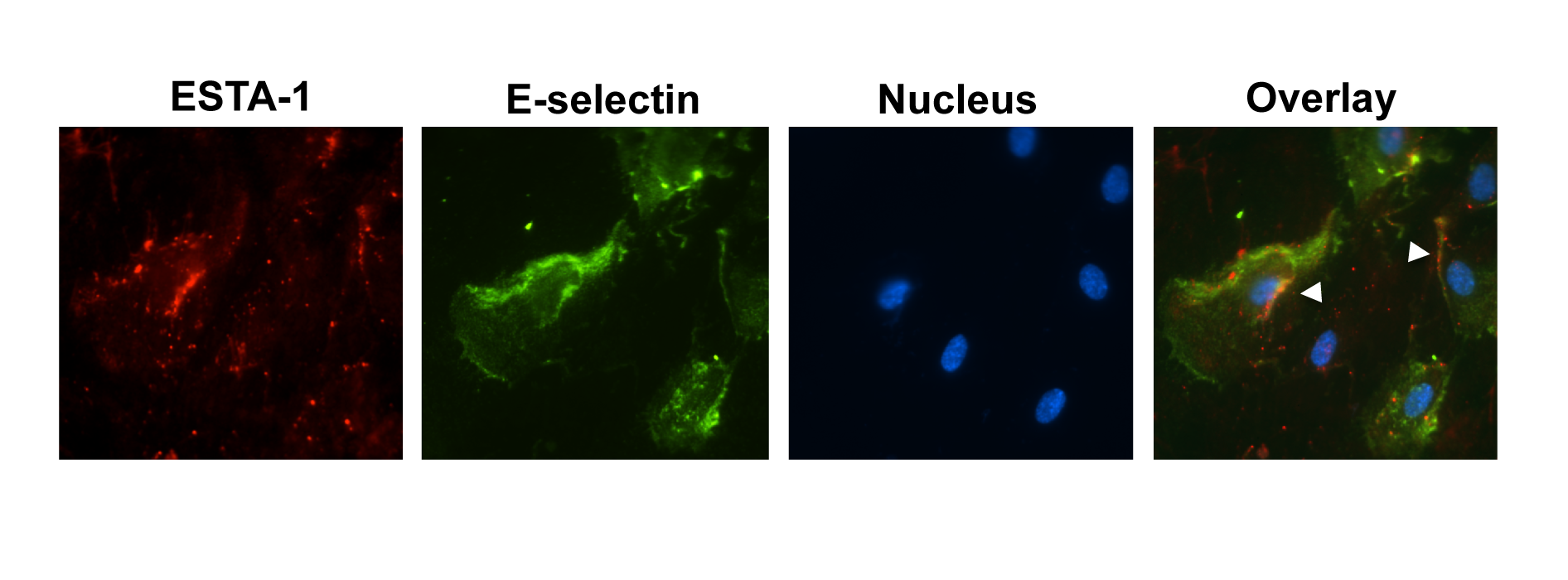

Supplement: Figure S6 — Colocalization of E-selectin expression and ESTA-1 binding to ES-Endo. ES-Endo cells were treated with doxycycline (2000 ng/ml) and analyzed for ESTA-1 binding and E-selectin expression using immunofluoroscence. Blue, Hoescht 33342; Red, Cy3-labeled ESTA-1; Green, E-selectin. (0.60 MB TIF) [file pone.0013050.s007.tif]
